# Real-World Experience With a Large Bore Vascular Closure Device During TAVI Procedure: Features and Predictors of Access-Site Vascular Complications

**DOI:** 10.3389/fcvm.2022.832242

**Published:** 2022-02-28

**Authors:** Giulia Masiero, Livio D'Angelo, Luca Nai Fovino, Tommaso Fabris, Francesco Cardaioli, Giulio Rodinò, Alice Benedetti, Mauro Boiago, Saverio Continisio, Carolina Montonati, Tommaso Sciarretta, Vittorio Zuccarelli, Andrea Scotti, Giulia Lorenzoni, Andrea Pavei, Massimo Napodano, Chiara Fraccaro, Sabino Iliceto, Alfredo Marchese, Giovanni Esposito, Giuseppe Tarantini

**Affiliations:** ^1^Department of Cardiac, Thoracic, Vascular Sciences and Public Health, University of Padua, Padua, Italy; ^2^Unit of Cardiology, GVM Care and Research, Anthea Hospital, Bari, Italy; ^3^Divisions of Cardiology and Cardiothoracic Surgery, Department of Advanced Biomedical Sciences, Federico II University, Naples, Italy

**Keywords:** transfemoral transcatheter aortic valve implantation (TF-TAVI), valve academic research consortium (VARC), vascular closure device (VCD), multidetector computed tomography (MDCT), common femoral artery (CFA), vascular complications

## Abstract

**Backgrounds:**

Among vascular closure devices (VCDs), the novel collagen plug-based MANTA VCD is the first designed for large bore percutaneous access. We aimed to assess the features and predictors of access-site vascular complications in an unselected trans-femoral transcatheter aortic valve replacement (TF-TAVR) population.

**Methods:**

Patients undergoing large bore arteriotomy closure with 18F MANTA VCD following TF-TAVR at a large tertiary care center from September 2019 to January 2021 were prospectively analyzed. Primary Outcome was the MANTA VCD access-site-related complications according to Valve Academic Research Consortium-3 (VARC) definitions. Its incidence and predictors were evaluated.

**Results:**

Eighty-eight patients (median age 82 years, 48% male, 3.3 median Society of Thoracic Surgeons score) undergoing TF-TAVR were included, mostly (63%) treated with a self-expandable device and with outer diameter sizes varied from 18F to 24-F. MANTA VCD technical success rate was 98%, while 10 patients (11%) experienced MANTA VCD access-site vascular complications which included 8% of minor complications and only to 2% of major events resulting in VARC type ≥2 bleeding. Vessel occlusion/stenosis (60%), perforation (20%), and pseudoaneurysm/dissection/hematoma (20%) occurred, but all were managed without surgical treatment. Independent predictors of failure were age (*p* = 0.04), minimum common femoral artery diameter (CFA) (*p* < 0.01), sheath-to-femoral-artery ratio (SFAR) (*p* < 0.01), and a lower puncture height (*p* = 0.03). A CFA diameter <7.1 mm with a SFAR threshold of 1.01 were associated with VCD failure.

**Conclusions:**

In a more comers TF-TAVR population, MANTA VCD was associated with reassuring rates of technical success and major access-site vascular complications. Avoiding lower vessel size and less puncture site distance to CFA bifurcation might further improve outcomes.

## Introduction

Catheter-based treatment strategies for complex cardiovascular disease requiring large bore femoral vascular access, such as trans-femoral transcatheter aortic valve replacement (TF-TAVR), markedly increased over the last decades (1). Nevertheless, access site vascular and bleeding complications still impact peri-procedural morbidity and mortality (2, 3). Newer iterations of TAVR devices and development of percutaneous vascular closure devices (VCDs) have contributed in lowering the incidence of such adverse events. However, percutaneous large bore arteriotomy closure with the most used suture-based closure devices remained associated with a 5–20% of complication rates (4, 5). The MANTA VCD (Teleflex Inc., Pennsylvania, USA) is the first commercially available collagen-based technology dedicated for large bore arteriotomy closure approved by the United States Food and Drug Administration (FDA) (6, 7). A reliable safety and efficacy have been well established in selected TAVR populations, but comparative studies with conventional suture-based devices have provided heterogenous results (8–12). Therefore, we sought to evaluate MANTA VCD performance for large bore arteriotomy closure in an unselected single-center TF-TAVR population, with a focus on access-site vascular complications rates and predictors.

## Methods

### Study Population

The ongoing Padua University REVALVing Experience (PUREVALVE) registry has prospectively collected data on all patients undergoing TAVR in this large tertiary care center since June 2007. For the purpose of this analysis, all consecutive patients undergoing large bore arteriotomy closure with MANTA VCD following TF-TAVR from September 2019 to January 2021 were included. No selection process for using or excluding the MANTA device was applied, identifying a so-called “more comers” population. Indications for TAVR, approach, and prosthesis choice were based on the Heart Team decision. Nonetheless, the Heart Team discussion did not influence the decision-making process on MANTA or other VCD. All subjects gave their informed consent for both TAVR and clinical data collection. Baseline and periprocedural characteristic and clinical outcomes data were collected up to 30-days follow-up and inserted in a dedicated dataset. The study was conducted according to the principles of the Declaration of Helsinki and Good Clinical Practice.

### Procedures

According to renal function, all patients underwent vascular ultrasound, multidetector computed tomography (MDCT), and/or angiography evaluation prior to TAVR to determine any significant vascular pathology in the planned femoral access site. Chronic anticoagulation therapy was interrupted at least 24 h prior to interventions. Procedures were performed by three different operators (none of them having prior MANTA deployment experience) and the implanted valves included both balloon and self-expandable bioprosthesis. The analysis also included the roll-in-cases, defined as the first ten consecutive cases per operator (9). Only an 18F MANTA VCD was used to accommodate 15-F to 21-F sheaths, with a maximum outer diameter (OD)/ profile of 24-F. A description of MANTA VCD characteristics and deployment steps were described in detail elsewhere (6, 7). In brief, prior to large sheath insertion, arteriotomy depth is determined with a depth locator with centimeter markers. After successful valve deployment, the large sheath is exchanged with the dedicated MANTA VCD sheath combined with a delivery unit which comprise a bioresorbable toggle and a hemostatic collagen plug. The system is then withdrawn over the wire to the labeled arteriotomy depth and the toggle is released inside the vessel by turning the lever of the handle and further withdraw the handle. Finally, the collagen plug is delivered outside the vessel by sliding down the tamper tube and cutting the suture at skin level (6, 7). Angiography-guided target femoral artery puncture, with contralateral injection, was mandatory, but no contralateral protection devices were used (e.g., 0.018 wire, balloon-assisted closure technique). A systolic blood pressure <180 mmHg was always achieved prior to arteriotomy closure and protamine was administered after angiographic confirmation of successful MANTA device deployment. Any additional vascular access site was closed using standard arteriotomy closure techniques. Post-procedural vascular ultrasound or multidetector computed tomography (MCDT) were not performed routinely but are mandatory in case of complications.

### Target Femoral Access Data

Pre-procedural MCDT and intra-procedural angiography analysis were performed in order to evaluate the following: the common femoral artery (CFA) and superficial femoral artery calibers, presence, site (anterior, posterior, lateral, and medial) and extension (crescent, spot, horseshoe, circumferential) of calcifications of the iliofemoral axis and of the puncture site (PS), and puncture height which is defined as the distance between the PS relative to the CFA bifurcation at the angiographic evaluation. Maximum sheath diameter, arteriotomy depth measured by the dedicated MANTA locator, and the sheath-to-femoral artery ratio (SFAR, defined as the ratio between the OD and the femoral artery minimal luminal diameter of the sheath) were also collected. All measurements were performed by two experienced structural interventional cardiologists (GM and LDA) who were unaware of patient clinical data.

### Clinical Outcomes Definitions

The MANTA VCD technical success was defined as the achievement of target arteriotomy closure without the use of bailout surgery or endovascular procedures (alternative to adjunctive endovascular ballooning), adapted from the Valve Academic Research Consortium-3 consensus document (VARC-2) for TAVR (13). The primary outcome was the occurrence of any minor or major primary access site vascular complications. In addition, all single minor and major vascular and bleeding complications were collected, both related to the primary and any accessory arterial vascular access site and complied with the VARC-3 criteria (11). Moreover, mortality, stroke, myocardial infarction, acute kidney injury, sepsis, and local femoral target side infection rates up to 30-days follow-up were also evaluated.

### Statistical Analysis

For the purpose of this analysis, we compared patients with vs. without MANTA-access vascular complications. Baseline and procedural characteristics are described with mean ± standard deviation (SD) or medians and 1st and 3rd interquartile ranges (IQRs) for continuous variables and percentages for discrete variables. Continuous variables were compared with one-way ANOVA and Student's unpaired *t*-test (parametric test) or the Kruskal-Wallis test and Wilcoxon rank-sum test (non-parametric test) between three and two groups, respectively. Normal distribution was evaluated with the Shapiro-Wilk test. Categorical variables (as frequencies or percentage) were compared with the χ^2^ test or the Fisher exact test, as appropriate. Logistic regression analysis was used to estimate possible MANTA-access vascular adverse events. All the covariates significantly associated with the outcome of interest at univariate analysis (*p* < 0.05 for model inclusion and *p* > 0.10 for exclusion) and those considered clinically relevant (as the presence of vascular ultrasound stenosis, claudication, vessel calcification, both extension and location, the minimum diameter of CFA or PS measured at MDCT and angiographically, the intraprocedural fluoroscopic calcium evaluation, the PS height relative to FCA bifurcation, and SFAR) were included. Results were reported as odds ratios (OR) and 95% confidence intervals (CI). For all analyses, a two-sided *p* < 0.05 was considered to be significant.

Taking into account all the independent predictors, Youden's index was used to identify the threshold of those variables to best forecast MANTA-access complications. The receiver-operating characteristics curve (ROC) and the related area under the curve (AUC) were reported for the identified thresholds. The analyses were performed using R software (version 4.0.2) with the rms and pROC packages (14–16).

## Results

### Patients and Procedures

**Figure 1:**
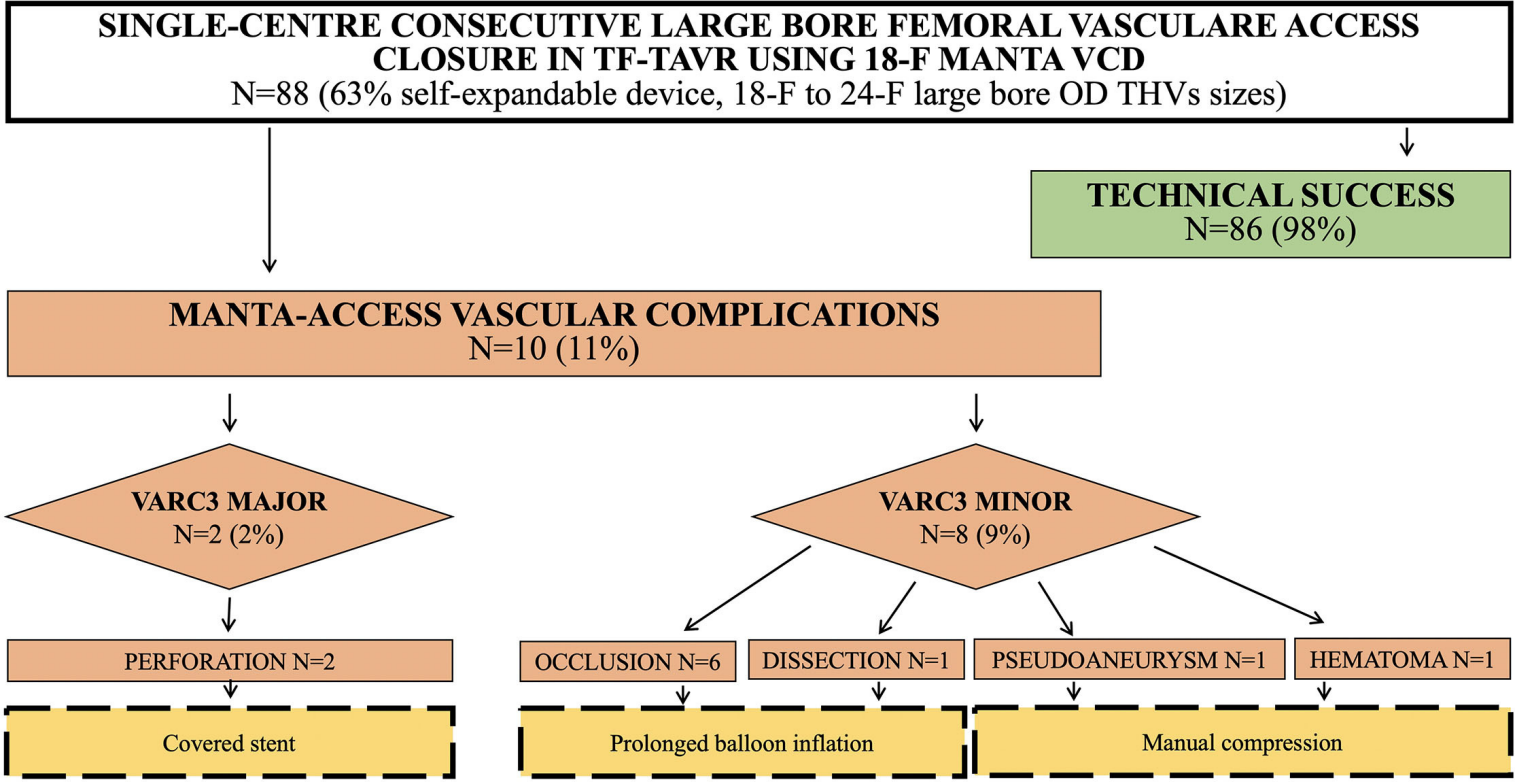
Study flow-chart of included patients undergoing Transfemoral Transcatheter Aortic Valve Implantation (TF-TAVR). TF-TAVR, Transfemoral Transcatheter Aortic Valve Implantation; VCD, vascular closure device; F, French; OD, outer diameter; THV, transcatheter heart valve; VARC, valve academic research consortium.

**Figure 2:**
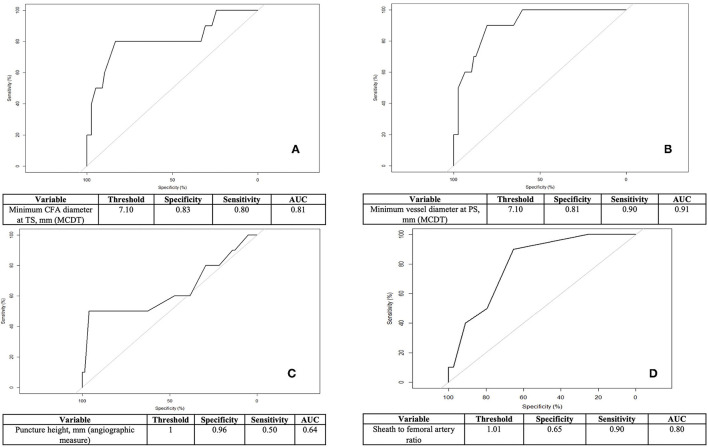
Independent predictors of MANTA-access vascular complications: receiver-operating characteristic (ROC) curve of their identified threshold to predict adverse events. **(A)** Minimum common femoral artery (CFA) diameter at target side (TS) at multidetector computed tomography (MCDT) evaluation (moderately accurate discriminant). **(B)** Minimum vessel diameter at puncture site (PS) at MCDT evaluation (highly accurate discriminant). **(C)** Puncture height relative to CFA bifurcation at angiographic measure (low accurate discriminant). **(D)** Sheath to femoral artery ratio (highly accurate discriminant).

A total of 88 consecutive patients undergoing TF-TAVR treated with 18F MANTA large bore arteriotomy closure were included in our analysis (Figure 1). Baseline characteristics of patients with and without MANTA access vascular complications are presented in Table 1. The median age was 82 (IQR 79–86 years), with significantly older patients in the complications group. Men represented 48% of the entire cohort with a median body mass index (BMI) of 27 (17–20). Comorbidities were similar between the two groups. Pre-procedural imaging and peri-procedural characteristics of patients are shown in Table 1. Right CFA was the target access in the vast majority of patients (92%) and self-expandable valve was implanted in nearly two thirds of the overall population (63%). The OD of the sheath varied from 18F to 24-F (Table 2). Patients in the main-access adverse events group had a significantly smaller CFA lumen at the MCDT evaluation, both the minimum [respectively, 6.6 (6.2; 7.0) vs. 7.9 (7.3; 8.7), *p* < 0.01] and the maximum [7.4 (7.0; 8.9) vs. 8.7 (8.0; 9.6), *p* = 0.02] diameter (Figure 2), and a significantly greater SFAR [1.15 (1.07; 1.2) vs. 93 (0.8; 1.03), *p* < 0.01] than those who did not experience complications. No differences were found in terms of the presence of stenosis of the target ileofemoral artery at the ultrasound assessment, the presence and location of calcification (both of the target ileofemoral artery and of the PS), the puncture height, and the grading of calcification (both at MCDT and at angiographic evaluations).

**Table 1:** Baseline, pre-procedural imaging, and peri-procedural characteristics of patients.

**Variable**	**No Manta-access vascular complications group (*N =* 78)**	**Manta-access vascular complications group (*N =* 10)**	**Overall population** **(*N =* 88)**	***P*-value**
**Baseline characteristics**				
Age, years	82.0 [78.3; 85.0]	85.0 [83.3; 86.8]	82.0 [79.0; 86.0]	0.023
Male gender	39 (50%)	3 (30%)	42 (48%)	0.233
Height, cm	166 [160; 172]	160 [157; 164]	165 [159; 172]	0.167
Weight, kg	75 [64; 82.7]	64.5 [56.7; 72.2]	72.5 [63; 82]	0.098
BMI, Kg/m^2^	27.0 [24.0; 29.0]	26.5 [23.3; 27.0]	27.0 [23.7; 29.0]	0.387
Obesity	17 (48%)	2 (20%)	19 (22%)	0.897
Hypertension	60 (77%)	10 (100%)	70 (80%)	0.089
Diabetes mellitus	27 (35%)	2 (20%)	29 (33%)	0.355
Smoking	18 (23%)	2 (20%)	20 (23%)	0.827
Dyslipidemia	38 (49%)	7 (70%)	45 (51%)	0.205
CRF	27 (35%)	6 (60%)	33 (38%)	0.119
History of CAD	39 (50%)	3 (30%)	42 (48%)	0.233
History of AF	25 (32%)	4 (40%)	29 (33%)	0.615
OAC use	25 (32%)	4 (40%)	29 (33%)	0.615
History of claudication	3 (4%)	0 (0%)	3 (3%)	0.528
Peripheral vascular disease	6 (8%)	1 (10%)	7 (8%)	0.8
STS score	3.3 [2.4; 4.2]	3.2 [2.8; 4.5]	3.3 [2.4; 4.4]	0.57
**Pre-procedural imaging and peri-procedural characteristics**				
Antiplatelet drugs use^*^:				0.724
None	2 (3%)	0 (0%)	2 (2%)	
Aspirin	17 (22%)	1 (10%)	18 (20%)	
Clopidogrel	10 (13%)	1 (10%)	11 (12%)	
DAPT	49 (63%)	8 (80%)	57 (65%)	
Presence of stenosis at target ileo-femoral artery (Ultrasound assessment)	3 (4%)	1 (10%)	4 (5%)	0.379
Max CFA diameter at TS, mm (MCDT measure)	8.7 [8.0; 9.6]	7.4 [7.0; 8.9]	8.6 [7.8; 9.4]	0.023
Min CFA diameter at TS, mm (MCDT)	7.9 [7.3; 8.7]	6.6 [6.2; 7.0]	7.9 [7.2; 8.6]	0.001
Max SFA diameter at TS, mm (MCDT)	6.4 [5.8; 7.0]	5.6 [4.7; 5.9]	6.3 [5.8; 6.9]	0.003
Min SFA diameter at TS, mm (MCDT)	5.9 [5.3; 6.5]	5.2 [3.9; 5.7]	5.6 [5.2; 6.5]	0.021
Max vessel diameter at PS, mm (MCDT)	8.5 [7.8; 9.6]	7.2 [6.7; 8.0]	8.4 [7.6; 9.5]	0.004
Min vessel diameter at PS, mm (MCDT)	7.9 [7.2; 8.7]	6.3 [6.1; 6.9]	7.8 [7.0; 8.6]	<0.001
Presence of target ileo-femoral artery calcification (MCDT)	58 (74%)	9 (90%)	67 (76%)	0.275
Calcification at PS (MCDT):	40 (51%)	7 (70%)	47 (53%)	0.264
No/Mild calcifications at PS	55 (71%)	5 (50%)	60 (68%)	0.19
Moderate/Heavy calcifications at PS	23 (29%)	5 (50%)	28 (32%)	0.19
Site of calcification at PS (MCDT):				0.096
Anterior	8 (20%)	1 (14%)	9 (19%)	
Medial	3 (8%)	0 (0%)	3 (6%)	
Posterior	29 (72%)	5 (71%)	34 (72%)	
Lateral	0 (0%)	1 (14%)	1 (2%)	
Calcification at PS (angiographic measure):				0.657
No/Mild	69 (88%)	8 (80%)	77 (88%)	
Moderate/Severe	9 (12%)	2 (20%)	11 (12%)	
Puncture height, mm (angiographic measure)	1.5 [1.1; 2.2]	0.7 [0.0; 2.0]	1.5 [1.0; 2.2]	0.152
Sheath size, F	14.0 [14.0; 16.0]	14.0 [14.0; 15.5]	14.0 [14.0; 16.0]	0.473
**Valve type:**				0.379
Balloon-expandable	29 (37%)	3 (30%)	32 (36%)	
Self-expandable	49 (63%)	7 (70%)	56 (64%)	
Right target vascular side	71 (91%)	10 (100%)	81 (92%)	0.323
SFAR	0.93 [0.8; 1.03]	1.15 [1.07; 1.2]	0.95 [0.85; 1.1]	<0.001

*BMI, body mass index; CRF, chronic renal failure (defined by GFR <60 ml/min/1.73m2 calculated with Cockcroft-Gault formula); CAD, coronary artery disease (defined as critical coronary vessel disease: left main stenosis >50%, other coronary vessel stenosis >70% in vessel major then 2 mm); AF, atrial fibrillation; OAC, oral anti coagulation; STS, society of thoracic surgeons. Obesity defined as BMI > 30; peripheral vascular disease defined as the presence of stenosis > 30% at the Ultrasound assessment or history of claudication*.

*DAPT, dual antiplatelet therapy; Max, maximum; Min, minimum; CFA, common femoral artery; MDCT, multidetector computed tomography; SFA, superficial femoral artery; PS, puncture site; TS, target side; F, French; SFAR, sheath-to-femoral artery ratio*.

*Presence of stenosis at target ileo-femoral artery at the Ultrasound assessment defined as stenosis > 30%. MDCT calcification grading: No/Mild (<1 cm, <180°) or Moderate/Heavy (>1 cm and/or >180°). Angiographic calcification grading: none, mild (some calcification), moderate (the course of the artery can be seen without injection of contrast dye) or severe (heavily calcified femoral arteries). Puncture height was defined as the distance between the puncture site relative to the CFA bifurcation at the angiographic evaluation. SFAR was calculated as the ratio between the sheath's outer diameter (mm) and the femoral artery minimal luminal diameter (mm)*.

*Values are n (%) or median IQR*.

* *Started before target procedure*.

**Table 2:** Device and sheath size.

**Valve**	**Valve size**	**Number of patients**	**Sheet**	**ID-F**	**ID mm**	**OD-F**	**OD mm**
Edwards Sapien 3 Ultra	23	14	14F eSheaths	14	4,7	23	7,64
Edwards Sapien 3 Ultra	26	10	14F eSheaths	14	4,7	23	7,64
Edwards Sapien 3 Ultra	29	8	16-F eSheaths	16	5,3	24,3	8,18
CoreValve PRO	23	3		16	5,3	20	6,7
CoreValve PRO	26	8		16	5,3	20	6,7
CoreValve PRO	29	4		16	5,3	20	6,7
Portico	27	4	FlexNav Large	15	5	19	6,3
Portico	29	4	FlexNav Large	15	5	19	6,3
Symetis Acurate S	S	7	iSleeve	14-21	4,7-7	18-24	6-7,9
Symetis Acurate M	M	12	iSleeve	14-21	4,7-7	18-24	6-7,9
Symetis Acurate L	L	4	iSleeve	14-21	4,7-7	18-24	6-7,9
Lotus	23	4	Small	20,1	6,7	22,5	7,95
Lotus	25	4	Large	21,3	7	23,7	7,41
Lotus	27	2	Large	21,3	7	23,7	7,41

*ID, internal diameter; OD, outer diameter; F, French*.

### Clinical Outcomes

The MANTA VCD was deployed successfully in all but two patients with a technical success rate of 98%.

Clinical outcomes according to VARC-3 definitions were listed in Table 3. Overall, 21 patients experienced vascular complications. Of these, 10 patients (11%) had primary access-related complications and 11 patients (13%) had adverse events related to the accessory arterial vascular access sites. Among the former 10 patients (Figure 1), 2 of them (2%) experienced major primary access complications resulting in VARC type ≥2 bleeding, while the remaining 8 (9%) had minor vascular complications without clinical impairment. In particular, six (60%) had occlusion or major stenosis, two (20%) had vessel perforation, one (1%) had pseudoaneurysm, one (1%) had dissection, and one (1%) had hematoma. Six patients were treated with prolonged balloon inflation, two patients received endovascular stenting, and two were managed with manual compression. No surgery was needed. All MANTA-access adverse events were diagnosed peri-procedurally except two patients who suffered incomplete arteriotomy closure complicated by a pseudoaneurysm and a local hematoma that was identified on day 2. Overall, vascular complications were associated with VARC type ≥2 bleeding in three cases (3%), two of which were attributable to the MANTA VCD failure and required packed red blood cells transfusions.

**Table 3:** Clinical outcomes up to 30-day follow-up.

**Variable**	***N =* 88**
Manta technical success	86 (98%)
All vascular complication	21 (24%)
Manta-access related complications:	10 (11%)
Major vascular complications	2 (2%)
Minor vascular complications	8 (9%)
**Manta-access vascular complications type:**	
Occlusion/Stenosis	6 (7%)
Perforation	2 (2%)
Dissection	1 (1%)
Pseudoaneurysm	1 (1%)
Hematoma	1 (1%)
Manta-access vascular complications treated with PRBCs	2 (2%)
**Manta-access vascular complications treatment:**	
Manual compression	2 (2%)
Prolonged balloon inflation	6 (7%)
Stenting	2 (2%)
Surgical repair	0 (0%)
Non Manta-access related complications:	11 (13%)
Major vascular complications	1 (1%)
Minor vascular complications	10 (11%)
**Non Manta-access vascular complications type:**	
Pseudoaneurysm	4 (5%)
Hematoma	11 (13%)
**Overall bleeding:**	
VARC Type 3–4	2 (2%)
VARC type 2	1 (1%)
VARC type 1	12 (14%)
Mortality	0 (0%)
Stroke	1 (1%)
Myocardial infarction	0 (0%)
Acute kidney injury	0 (0%)
Sepsis	0 (%)
Manta-access local infection	0 (0%)

*All definitions were complied with the Valve Academic Research Consortium-3 consensus document (VARC-3) for TAVR (13)*.

*PRBCs, packed red blood cells*.

No death, myocardial infarction, AKI, sepsis, and femoral target side local infection rates up to 30-days follow-up occurred. One patient had a hemorrhagic stroke.

### Predictors of MANTA VCD Failure

Logistic regression analysis for predictors of MANTA-access vascular complications is presented in Table 4. Independent predictors were age [odds ratio: 1.4, 95%, confidence interval (1.1; 4.8), *p* = 0.04], the roll-in case performance (OR 4.2, 95% CI 1.1-17.4, *p* = 0.048), the minimum femoral artery diameter both at TS and PS [OR: 6.7, 95% CI (2.1; 25.1), *p* = 0.002 and OR: 8.8, 95%, CI (3.5; 50.1), *p* = 0.001, respectively], SFAR [OR: 1.9, 95%, CI (1.2; 3.1), *p* = 0.006], and a lower puncture height relative to the femoral bifurcation [OR:0.4, 95%, CI (0.2;0.9), *p* = 0.03]. The identified threshold of the minimum CFA diameter to best predict vascular adverse events was 7.1 mm with a SFAR threshold of 1.01. Using this cut-off point for the ROC, both are identified as intermediate to high accurate discriminant of device failure (respectively with an AUC of.91 and 0.81). Otherwise, a puncture <1 mm or below the CFA bifurcation resulted in a less powerful discriminant of MANTA-access vascular complications.

**Table 4:** Predictors of Manta-access vascular complications.

**Variable**	**OR [CI 95%]**	***p*-value**
Age	1.43 [1.11; 4.78]	0.043
Male gender	0.43 [0.11; 1.78]	0.243
BMI	0.67 [0.28; 1.57]	0.356
Oral anticoagulation use	1.41 [0.37; 5.46]	0.616
Peripheral vascular disease	1.93 [1.21; 3.09]	0.800
Presence of stenosis at target ileo-femoral artery (Ultrasound assessment)	2.78 [0.26; 29.61]	0.3975
History of claudication	0.01 [Not estimable]	0.8090
Minimum CFA diameter at TS (MCDT)	6.67 [2.04; 25.1]	0.0019
Minimum vessel diameter at PS (MCDT)	8.84 [3.45; 50.1]	0.0011
Presence of at least moderate target ileo-femoral artery calcification (MCDT)	2.39 [0.63; 9.06]	0.199
Site of calcification at PS (MCDT)	0.73 [0.07; 7.13]	0.783
Presence of at least moderate calcification at PS (angiographic measure)	1.89 [0.33; 10.77]	0.4719
Puncture height (angiographic measure)	0.43 [0.19; 0.93]	0.0333
Sheath size (F)	0.69 [0.31; 1.57]	0.3812
SFAR	1.93 [1.21; 3.09]	0.006
Roll-in case	4.17 [1.1; 17.41]	0.048

*BMI, body mass index; CFA, common femoral artery; PS, puncture site; TS, target side; MDCT, multidetector computed tomography; F, French; SFAR, sheath-to-femoral artery ratio*.

## Discussion

This is a prospective single-center analysis on the use of the 18F MANTA VCD for large bore arteriotomy closure in a more comers population treated by TF-TAVR that focus on the features and predictors of access-site vascular complications. The main findings are: (1) the use of MANTA device was associated with favorable technical success and major primary access site vascular complications rates; (2) the most frequently encountered vascular complication with MANTA VCD was target vessel occlusion/stenosis; and (3) independent predictors of the primary access complications were age, small femoral artery diameter, larger SFAR, and a lower puncture height.

Vascular complications at the puncture site after TAVR negatively affect clinical outcomes (2, 13). Despite wide adoption of VCD, contemporary trials still reported major vascular complications after TAVR in more than 5% of patients, and two thirds of them were secondary to failed arteriotomy closure by suture-based device (1).

The dedicated collagen plug-based MANTA VCD is a novel method for large caliber arteriotomy closure, with promising safety and efficacy profile in highly selected patients with major and minor vascular complication rates ranging between 2–5% and 0–8%, respectively (6–10). In latest experiences, including more comers patients at intermediate to high surgical risk treated with TAVR and using 14F/18F MANTA VCD, the adverse vascular event rate was up to 14% (21–25). Moreover, comparative data with conventional suture-based devices have showed discordant results, highlighting a higher than anticipated rate of MANTA-access vascular events (up to 19%) in the early randomized studies (8–12).

In our analysis focused on intermediate/low risk TAVR-treated patients, we confirmed a high arteriotomy closure success rate (98%) by the use of MANTA device, extending these results to patients also excluded in early studies as those with obesity, excessive calcifications, and/or tortuosity of the ileo-femoral artery (6–10). The rate of overall MANTA-access vascular complications (11%) was in line with previous observational registries and early trials and is definitely lower than in the largest randomized available experience mainly driven by a reduction of major events (8–12, 25). Moreover, we found a lower rate of surgical repair and major or life threatening bleedings due to MANTA failure as compared to registries including patients with similar vessel size, SFAR ratio, and severity of calcification (12, 23, 26). The systematic performance of ileo-femoral angiography to visualize the site of the primary puncture before and to assess possible vascular complications after the vascular closure permitted to promptly treat and avoid major events (17, 27). However, as being related to post-procedural morbidity, we should acknowledge a non-negligible rate of access-site minor vascular complications and minor bleedings. As speculated by Abdel-Wahab et al., the MANTA VCD might imply a misleading impression of early hemostasis followed by an insufficient manual compression without allowing easy rescue techniques (such as additional suture-based devices) due to the retrieval of the vascular wire before deployment (12).

To note, the main observed reason of MANTA-access adverse vascular events was CFA occlusion secondary to the toggle misplacement in femoral arteries with smaller caliber. Different from previous reports, we failed to find a significant increase in significant bleeding and pseudoaneurysm formation due to incomplete apposition of the collagen-based plug (12, 18, 23, 26). We cannot exclude that this difference was related to the exclusive use of 18F device that matched more appropriately in our series the OD/profile of the implanted TAVR systems.

Related to the predictors of vascular complications, by our multivariate analysis, we found that in pre-procedural planning, it is important to accurately assess the size of the CFA with the expected SFAR for the chosen TAVR prosthesis. In particular, a minimum CFA of 7.1 mm and SFAR of 1.01 is essential to envisage an uneventful 18F MANTA implantation. During TAVR procedure, the other major predictor of the primary-access vascular complications is the height of implantation related to the CFA bifurcation. To note, when the minimum reference CFA caliber is met, the severity and location of vessel calcification do not seem to play a major role in device failure as shown by others (9, 10).

Finally, the inclusion of the roll-in-cases in our series negatively impact the occurrence of vascular adverse events, confirming the importance of the learning curve of an operator, which had previously been reported with suture-based but not collagen-based VCD (10, 19, 20, 23, 25). Despite the ease of use of the device, a growing experience might have an impact on vascular complication occurrence presumably because of improved patient-selection (e.g., avoiding smaller vessels) and the achievement of an adequate skills set of the operators (e.g., choosing higher puncture height).

### Study Limitations

For a thorough understanding of our results, we should acknowledge several limitations. This was a single-center registry including a relatively small number of patients with a non-randomized design. Moreover, the clinical outcomes were not adjudicated by an independent committee. Therefore, our results may be influenced by unknown confounding variables and be underpowered to distinguish other predictors of MANTA-access adverse vascular events. Nevertheless, our sample size is comparable to previous reports, and we added additional insights on a broader population treated by highly experienced TAVR operators.

Moreover, similar to previous analysis (11, 12, 25), only 18F MANTA was used for all patients due to cath lab availability. Therefore, our results have not to be generalized to 14F MANTA VCD.

Access technique (i.e., the lack of US guidance for CFA puncture) may have affected clinical outcomes. Furthermore, the lack of systematic post-procedural US assessment of TAVR access may have led to underreporting of vascular complications as pseudoaneurysms. Larger multi-center studies are warranted to accurately assess the impact of MANTA VCD usage, device failure, and clinical outcomes.

## Conclusion

This more comers experience with MANTA VCD during TF-TAVR procedure showed reassuring rates of technical success and major access-site vascular complications, with a non-negligible rate of minor vascular events. Avoiding lower vessel size, less PS distance to CFA bifurcation and performing a systematic ipsilateral ilio-femoral angiography before vessel puncture and after MANTA VCD deployment might further increase the rates of successful arteriotomy closure.

## Data Availability Statement

The data that support the findings of this study are available from the corresponding author, Giuseppe Tarantini, upon reasonable request.

## Ethics Statement

The studies involving human participants were reviewed and approved by the local ethical committee of Padua University Hospital. The patients/participants provided their written informed consent to participate in this study.

## Author Contributions

In particular, GT, GM, GL, and LD'A participated to the conception, design, analysis, and interpretation of data. All authors contributed to the drafting of the manuscript, its critical revision for important intellectual content, the final approval of the submitted text, agreed to be accountable for all aspects of the work in ensuring that questions related to the accuracy or integrity of any part of the work are appropriately investigated and resolved, and have contributed significantly to the submitted work.

## Conflict of Interest

GT reports honoraria for lectures/consulting from Medtronic, Edwards Lifesciences, Boston Scientific, GADA, and Abbott. The remaining authors declare that the research was conducted in the absence of any commercial or financial relationships that could be construed as a potential conflict of interest.

## Publisher's Note

All claims expressed in this article are solely those of the authors and do not necessarily represent those of their affiliated organizations, or those of the publisher, the editors and the reviewers. Any product that may be evaluated in this article, or claim that may be made by its manufacturer, is not guaranteed or endorsed by the publisher.

## Abbreviations

TF-TAVI: transfemoral transcatheter aortic valve implantation
VARC: valve academic research consortium
VCD: vascular closure device
MDCT: multidetector computed tomography
FDA: food and drug administration
CFA: common femoral artery
CT: computed tomography
IFU: instruction for use
BMI: body mass index
OD: outer diameter
PTA: percutaneous transluminal angioplasty
AKI: acute kidney injury
AVF: arteriovenous fistula
OR: odds ratio
CI: confidence interval
AUC: area under the curve
SFAR: sheath-to-femoral-artery ratio.
